# *crw1* - A Novel Maize Mutant Highly Susceptible to Foliar Damage by the Western Corn Rootworm Beetle

**DOI:** 10.1371/journal.pone.0071296

**Published:** 2013-08-09

**Authors:** Bala Puchakayala Venkata, Nick Lauter, Xu Li, Clint Chapple, Christian Krupke, Gurmukh Johal, Stephen Moose

**Affiliations:** 1 Department of Botany and Plant Pathology, Purdue University, West Lafayette, Indiana, United States of America; 2 USDA-Agricultural Research Service, Iowa State University, Ames, Iowa, United States of America; 3 Plants for Human Health Institute, North Carolina State University, Kannapolis, North Carolina, United States of America; 4 Department of Biochemistry, Purdue University, West Lafayette, Indiana, United States of America; 5 Department of Entomology, Purdue University, West Lafayette, Indiana, United States of America; 6 Department of Crop Sciences, University of Illinois at Urbana-Champaign, Champaign, Illinois, United States of America; China Agricultural University, China

## Abstract

Western corn rootworm (WCR), *Diabrotica virgifera virgifera* LeConte (Coleoptera: Chrysomelidae), is the most destructive insect pest of corn (*Zea mays* L.) in the United States. The adult WCR beetles derive their nourishment from multiple sources including corn pollen and silks as well as the pollen of alternate hosts. Conversely, the corn foliage is largely neglected as a food source by WCR beetles, leading to a perception of a passive interaction between the two. We report here a novel recessive mutation of corn that was identified and named after its foliar susceptibility to corn rootworm beetles (*crw1*). The *crw1* mutant under field conditions was exceptionally susceptible to foliar damage by WCR beetles in an age-specific manner. It exhibits pleiotropic defects on cell wall biochemistry, morphology of leaf epidermal cells and lower structural integrity via differential accumulation of cell wall bound phenolic acids. These findings indicate that *crw1* is perturbed in a pathway that was not previously ascribed to WCR susceptibility, as well as implying the presence of an active mechanism(s) deterring WCR beetles from devouring corn foliage. The discovery and characterization of this mutant provides a unique opportunity for genetic analysis of interactions between maize and adult WCR beetles and identify new strategies to control the spread and invasion of this destructive pest.

## Introduction

Corn rootworms are among the most serious insect pests of corn in the United States (U.S.) and have recently been introduced to the European Union (E.U.) as well [1]. The corn rootworm or *Diabrotica* complex consists of four economically important species: the western corn rootworm *Diabrotica virgifera virgifera* LeConte (WCR), northern corn rootworm *Diabrotica barberi* Smith (NCR), Mexican corn rootworm *Diabrotica virgifera zeae* Krysan and Smith (MCR), and southern corn rootworm *Diabrotica undecimpunctata howardi* Barber (SCR). Very few insect pests have presented more challenges to the U.S. agriculture than the corn rootworm complex [2]. Historically, the extent of insecticidal use for *Diabrotica* corn rootworms far exceeds that for any other U.S. agricultural pest [3]. The most devastating of the corn rootworm complex is WCR, which overwinters as eggs that hatch into larvae the following spring [1]. After three larval instars, the WCR larvae pupate briefly prior to the emergence of adult beetles in early summer [4]. The larval stage which feeds on roots of corn and a few other grass species is significantly more damaging than the adult beetle [5]–[7]. Extensive larval feeding can interfere with the ability of plants to absorb water and nutrients causing direct losses in grain fill in addition to plant lodging, complicating harvest activities [8]. Damage caused by larval feeding might also predispose roots to infection by root and stalk rot fungi [9]. Overall, WCR damage results in yield losses and control costs that long exceeded $1 billion per year [10], earning it the nickname of ‘billion dollar bug’ (www.GMO-Safety.eu.). Crop rotation and use of insecticides (soil and foliar) were the primary management strategies in North America throughout the last half of the 20^th^ century [11], [12]. However, the emergence of WCR variants with either extended diapause, a lack of fidelity to corn for oviposition, the evolution of counter-resistance to insecticides, and larger plantings of continuous corn have each contributed to significant range expansion of this insect pest [1]–[2], [13]. The reduced effectiveness of crop rotation has also significantly increased growers’ reliance on soil insecticides for first-year corn [14]. The commercial availability of rootworm-protected transgenic corn hybrids since 2003 has provided an effective alternative [15]. However, none of the transgenic events currently registered for WCR control expose larvae to toxin levels considered to be a high dose, leading to concerns about resistance development [16]. These concerns have been further elevated by recent laboratory and field demonstrations of rapid response to selection in the absence of mating with unexposed beetles [17], [18]. An alternate, or complementary, WCR management strategy could involve the use of native plant resistance. Despite extensive screening efforts, corn germplasm with robust resistance to WCR has not yet been identified [1], [19].

While most WCR management strategies focus upon reducing larval feeding upon roots, a sustainable alternative could involve managing the population level and behavior of adult WCR, which is a beetle. The WCR beetles are polyphagous i.e. derive nourishment from multiple sources including corn pollen and silks along with pollen of alternate hosts, but very rarely from corn leaves [20], [21]. The WCR beetles display very low preference for late vegetative or reproductive stage corn foliage [22], [23], suggesting a passive interaction between WCR beetles and corn leaves [20]. Although corn germplasm with robust resistance to WCR larvae is lacking [1], observed variation in susceptibility to foliar feeding by the WCR beetle [24], indicates that corn foliage may have effective mechanisms for resistance.

In the present study we report the identification, phenotypic characterization, and genetic mapping of *corn root worm1 (crw1)*, a corn locus defined by its developmentally- dependent foliar susceptibility to WCR beetles. We demonstrate that the adult leaves of *crw1* mutants retain the cell wall biochemistry properties associated with juvenile epidermal cells, including toluidine blue-O (TBO) staining pattern, epidermal lobe formation and reduced accumulation of both hydroxycinnamic acids and lignin. Additionally, the fracture dynamics studies are indicative of reduced structural integrity of the adult mutant leaves. Furthermore, molecular mapping studies using simple sequence repeat (SSR) markers delineated the position of *Crw1* to chromosome 6. To our knowledge similar mutant phenotypes have not been previously reported in corn or any other grass species. The *crw1* mutant unveils a previously undescribed pathway(s) or mechanism(s) for native resistance to foliar feeding by this most devastating insect pest of maize.

## Materials and Methods

### Plant Materials and Growth Conditions

The maize (*Zea mays* L.) *crw1* mutant (*crw1)* was identified by Guri Johal in three separate F_2_ populations which shared a common pedigree. These were originally generated by Tom Brutnell’s laboratory to scatter *Ac* elements across the maize genome, and planted at University of Illinois in the summer of 2005. All field experiments for genetic analysis and phenotypic characterization of the *crw1* mutant were performed at Purdue Agronomy Center for Research and Education (ACRE) farm in West Lafayette, Indiana. Eighteen seeds per row were planted in 6 m rows space 0.76 m apart. For stagger planting experiments the wild type and *crw1* plants were planted in alternating rows at bi-weekly intervals starting from the end of May. This allowed us to have the wild type and *crw1* plants at various growth stages coinciding with the emergence of adult western corn rootworm (WCR) beetles (*Diabrotica virgifera virgifera* LeConte). All experiments were conducted using natural infestations since the ACRE farm has traditionally exhibited heavy WCR pressure.

The *crw1-4* mutant was identified by Steve Moose from M_2_ families derived from ethyl methanesulfonate mutagenesis of an inbred line that was homozygous for the *ramosa1-RS* mutation [25], on the basis of purple staining with TBO of 5-mm diameter circular punches obtained from adult leaves. The TBO staining was performed following a published protocol [26]. The *crw1-4* mutant allele was introgressed into the B73 and Mo17 inbred lines by six backcrosses followed by two generations of self-pollination to recover homozygous near isogenic lines. The B73 and Mo17 *crw1-4* near-isogenic lines were crossed to each other to generate hybrid seeds. The B73 × Mo17: *crw1-4* hybrid, along with a similarly-constructed near-isogenic hybrid for the *glossy15-H* mutation and the wild-type B73 × Mo17 hybrid, were each planted in adjacent field plots (5.3 m long and 0.76 m row spacing, 30 plants per row) at the Crop Sciences Research and Education Center at the University of Illinois during the 2008 growing season. The extent of foliar feeding damage was quantified using a rating system of 1 for no foliar feeding to 10 for full defoliation of the most distal 25 cm of the leaf subtending the ear node. Root damage ratings were collected from excavated roots that were soaked, washed and rated for damage using the 0–3 scale [27]. Grain yields were estimated by harvesting all ears from plots, weighing the shelled grain, and adjusting for moisture content measured by near-infrared spectroscopy on a Dickey- John Instalab 600 instrument.

Double mutant analyses of *crw1* were conducted by crossing the B73: *crw1-4* near-isogenic line to either a B73: *gl15-H* near-isogenic line, or the *brown midrib 1-4* mutant stocks (119F, 408E, 515D, 918B) available from the Maize Genetics Cooperation Stock Center (www.maizegdb.org). F_1_ plants were then self-pollinated and at least 100 F_2_ progeny planted in the field were phenotyped for TBO staining in adult leaves, leaf waxes and macrohairs, midrib color, and foliar damage from WCR beetles.

### Genetic Mapping of *crw1*

Following three backcrosses of the *crw1-4* mutation to the maize inbred line B73 and self-pollination, a population of 441 plants was scored for the *crw1* mutant phenotype by TBO staining of leaf 10. The population produced 323 wild-type plants that stained aqua with TBO and 118 *crw1-4* mutant plants that stained purple. Genomic DNA was isolated from three separate pools of 12 wild-type and 12 *crw1-4* mutant plants and each pool was screened by PCR amplification with a set of 383 simple sequence repeat (SSR) markers selected to be evenly distributed across the maize genetic map (available from Sigma-Aldrich and www.maizegdb.org). Eight markers located to chromosome six showed amplification patterns indicative of segregation distortion and hence potential linkage with *crw1*. Five of these markers (*bnlg161*, *gpc2*, *umc1753*, *bnlg1867*, *umc1229*) were then used to genotype 112 *crw1-4* mutant individuals. Genotyping data was used to estimate recombination frequencies between *crw1* and each of these markers.

### Toluidine Blue- O (TBO) Staining of Maize Epidermal Peels

Leaf discs from juvenile and adult leaf discs (defined here as leaves number 1–6 and 13 and above respectively, counting the first leaf to be initiated as leaf number 1) from nine plants each of *crw1* and wild-type were collected. These samples were fixed in 4% formaldehyde in PBS and 0.2% saponin (Sigma- Aldrich, St. Louis) pH 7, washed and incubated with 0.1% pectinolyase overnight [28]. The epidermis was then peeled from the rest of the leaf using a fine point forceps and incubated in 0.05% TBO pH 4.0 until evenly stained. The stained samples were washed and imaged using bright field optics (Olympus Vanox, Olympus Corporation, New Hyde Park, New York).

### Cryo-scanning Electron Microscopy

Two leaf strips, ½ cm wide by 1 cm long, were cut 5 cm from the apical and basal portions of juvenile (leaf number 3), transition (leaf number 7) and adult (leaf number 13) leaves from 9 plants each of *crw1* and wild-type. Sample pieces were mounted on a flat holder covered with double-coated carbon tape and cryo-adhesive. They were further secured using narrow carbon tape strips on each side of samples. The sample holder was plunged into liquid nitrogen slush. A vacuum was pulled and the samples were transferred to the Gatan Alto 2500 pre-chamber (cooled to ∼170°C). Samples were sublimated at −90°C for 7 min followed by sputter coating for 90 sec. with platinum. The sample holder was then transferred to the microscope cryo-stage (∼−130°C) for imaging. Samples were imaged using an FEI NOVA nano SEM field emission scanning electron microscope (FESEM, FEI Company, Hillsboro, Oregon) operated under high vacuum conditions using an Everhart-Thornley secondary electron detector and Through-the-Lens (TLD) high resolution detector. Parameters were 5 kV, Spot 3, 5 mm working distance, and magnifications of 150, 500, and 2000×.

### Uni-axial Tensile Stage Test

Uni-axial tensile test was performed using a GATAN (Warrendale, PA, USA) low force tensile stage with a 20 Newton load cell. Adult leaf (leaf number 13) samples from 9 plants each of *crw1* and wild-type were prepared by cutting 7 mm wide by 4 cm long leaf strips from a constant position along the leaf lamina. A 1 mm incision was made exactly at the midpoint of each leaf strip tested, using a razor blade mounted on a pre-fabricated metallic aid under a stereomicroscope. The samples were affixed between the clamps across the crossheads of the tensile stage. The tensile tests were conducted at a constant extension rate of 1 mm/min. The stage was set to a minimal extension and force at zero before each run. Two replicates from the same position constituted one sample and two samples, one each from the apex and base were taken from every leaf tested. The cryo system attached to the tensile stage enabled us to monitor the incipient crack propagation and SEM imaging of the fracture surface at the point of final failure.

### Analysis of Cell Wall Bound Hydroxycinnamic Acids

For cell wall preparation, whole plant leaf tissue from 3 biological replicates (3 plants/replicate) each of V3 and V8 stage *crw1* and wild-type were harvested and flash frozen in liquid nitrogen after removing the midribs. The leaf samples were grounded to a fine powder in liquid nitrogen and extracted with neutral phosphate buffer, 70% ethanol and acetone [29], [30]. Cell wall esterified phenolics were released by saponification with 1 M sodium hydroxide (NaOH) for 16 h at room temperature and the hydrolysis products were extracted in ethyl acetate and separated by reverse phase HPLC using detection at 280 and 320 nm.

### Lignin Analysis

Whole plant leaf tissue from 9 biological replicates (3 plants/replicate) each of V8 stage *crw1* and wild-type adult plants were harvested and flash frozen in liquid nitrogen after removing the midribs. Cell walls were prepared from these leaf samples as mentioned in the previous section. Two milligrams of the dried cell wall material was used for acetyl bromide-soluble lignin (ABSL) analysis following the published protocol [31], with slight modifications. Briefly, the cell wall material was dissolved in 100 μl freshly made acetyl bromide/acetic acid solution (20∶80, v/v) in a 2 ml volumetric flask. The solution was incubated at 50°C for 2 hr with vortexing every 15 min. The mixture was brought to room temperature by cooling on ice and lignin was extracted using a mixture of 400 μl 2 M NaOH and 70 μl 0.5 M hydroxylamine hydrochloride. The volume of the mixture was made up to 2 ml with glacial acetic acid and mixed thoroughly. The percentage of ABSL (% of ABSL) was determined by pipetting 200 μl of the solution into a UV-specific 96 well plate and read in an ELISA reader at 280 nm. Three readings were performed for every sample extracted and the proportion of ABSL lignin was determined by using 18.21 as an extinction coefficient.

## Results

### Discovery and Genetics of *crw1*

Approximately 2000 F_2_ families derived from *Ac* transposon-mediated mutagenesis of the maize inbred W22 [32] were planted at the University of Illinois in the summer of 2005 and visually examined for a diverse set of phenotypes at the post-flowering stage. Three independent F_2_ populations were identified that segregated for plants which displayed signs of insect herbivory. Only a subset of the individual plants within each of the affected families showed insect damage, and these were adjacent to plants with no foliar feeding, suggesting a segregating genetic basis for the phenotype. The absence of the insect causing the damage at this late stage of crop development precluded us from identifying the species responsible.

Additional seeds of the affected F_2_ populations were acquired and replanted at Purdue University’s Agronomy Center for Research and Education (ACRE) farm in West Lafayette, Indiana in 2006. Plants were checked regularly for germination, growth, development, and symptoms associated with biotic or abiotic stress. None of the plants in any of these F_2_ families exhibited any kind of abnormality during the first few weeks of growth. However, when plants reached V5–V6 stage, a few plants from each of the three populations came under attack by the WCR beetles. The number of beetles increased over time on these selected plants which led to their substantial defoliation. The rest of the plants in each F_2_ family remained largely untouched by the beetles. These results clearly indicated that genetic mutations led to exceptional foliar herbivory by the WCR beetles. To address whether these mutants were allelic, they were crossed with at least three wild-type plants within each F_2_ family and the resulting progenies were tested for susceptibility to WCR beetles. Roughly half of the plants in 15 of the 24 progenies thus produced displayed foliar susceptibility to WCR beetles. The appearance of WCR susceptible mutants in all possible mutations in a single locus. This locus has been designated *crw1*, for susceptibility to *c*orn *r*oot*w*orm*1*. These results also suggested that *crw1* was probably a recessive mutation. To test this hypothesis, one of the *crw1* alleles (*crw1-1*) was crossed to B73, a maize inbred often used for genetic and genomics studies. As expected, the resulting F_1_ hybrid was resistant to WCR, but the *crw1* mutant phenotype reappeared in the F_2_ population at a frequency consistent with inheritance as single locus recessive allele (17 mutants among 74 individuals, X^2^, *p*>0.05, 1 d.f.).

### Developmental Manifestation of *crw1*

**Figure 1:**
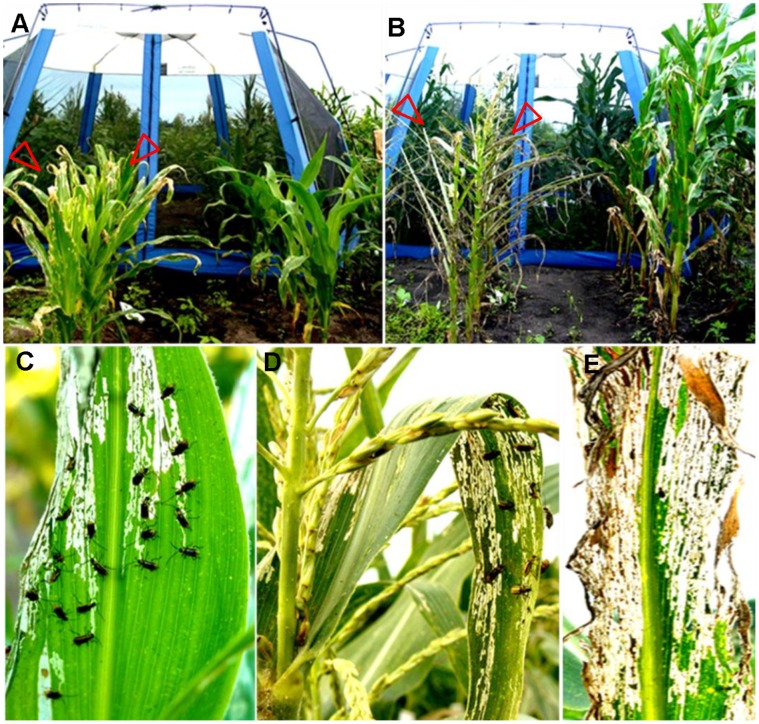
Phenotype of field grown *crw1*. **A**, Initiation of WCR beetle feeding of *crw1* around week 5–6 after planting (left, area between arrowheads). **B**, Once initiated, the WCR beetle feeding results in complete loss of foliage during the adult stage (left, area between arrowheads). The wild type (W22 inbred) on the other hand does not show WCR beetle damage at the same stages (A and B right). **C and D**, stripping of epidermal tissue on underside of leaves in a basipetal fashion in *crw1* by WCR beetles. **E**, characteristic “window pane” pattern resulting due to WCR feeding of *crw1* leaves.

An interesting feature of the *crw1* phenotype that we noticed was that significant WCR beetle damage did not start until the mutant reached the age of about 5–6 weeks. This could have been coincidental, given that our genetic nurseries are about 5–6 weeks old in late June or early July when the WCR beetles emerge. To examine whether the delayed manifestation of *crw1* susceptibility was merely due to the absence of WCR beetles during earlier stages of plant growth, *crw1* mutants (*crw1*) and their isogenic wild-types were planted at weekly intervals from the middle of May to the end of June. This planting scheme allowed the availability of *crw1* of all ages at the time of beetle emergence. It was found that regardless of the availability of mutant juvenile seedlings, the WCR beetles always preferred the foliage of *crw1* plants starting from V5–V6 stage (Fig. 1A, left panel). Interestingly, once the susceptibility to the WCR beetle became established in *crw1*, it lasted throughout the life of the mutant plants (Fig. 1B, left panel). In contrast, the wild-type isogenics lacking the *crw1* mutation remained resistant to WCR beetle damage throughout their lives (Fig. 1A and B, right panels).

Unusual susceptibility of *crw1* to WCR beetles enabled us to examine the feeding behavior of the WCR beetle. The beetle prefers to eat *crw1* leaves from below, the abaxial side (Fig. 1C). The damage often initiates near the tip of leaves first and proceeds largely downwards in a basipetal fashion (Fig. 1C and D). The WCR beetles scrape or chisel away all the leaf tissue from the upper epidermis, which is left intact. This results in transparent areas on the damaged leaves that have a “window pane” appearance (Fig. 1E). Being weak and flimsy post damage, the upper epidermis however withers away over time, giving the damaged plants a defoliated appearance with bare midribs (Fig. 1B, left panel). The entire foliage of *crw1* can be eaten away if the population level of the beetle is very high. However, if the beetle pressure is low, the damage to *crw1* is accordingly also low. In the absence of WCR beetles, or when plants are grown in a greenhouse, the mutant plants are almost indistinguishable from their wild type siblings in growth and vigor. Thus, the phenotype of *crw1* is conditional, being contingent on the presence of WCR beetles.

### *Crw1* is also Required for Biochemical and Morphological Features of Adult Leaf Epidermal Cell Identity

**Figure 2:**
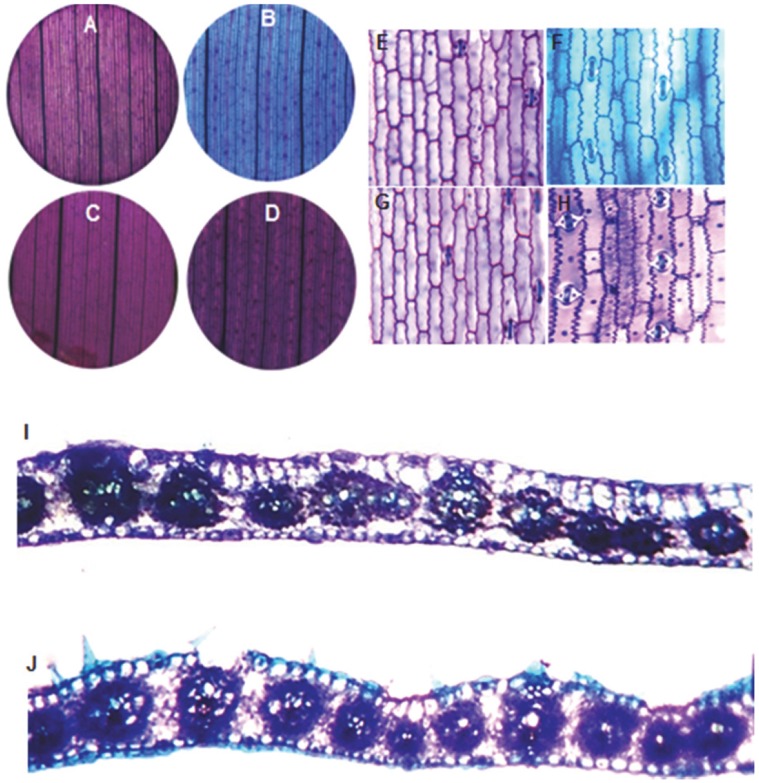
Representative TBO staining pattern of upper epidermis in the *crw1* mutant and wild-type leaves. Violet staining of upper epidermis from leaf 3 of wild-type (**A, E**), *crw1-4* mutant (**C**), and *crw1-1* mutant (**G**). **B and F**, aqua staining of wild type leaf 13. **D and H**, retention of violet staining in *crw1-4* (**D**) and *crw1-1* (**H**) leaf 13. **I-J**, cross section of *crw1-4* adult leaf 13 with all violet and no aqua staining (**I**) compared to aqua and violet staining (**J**) of wild-type leaf.

The unique phenotype of late-onset susceptibility to foliar feeding by WCR beetles was also observed to be associated with another maize mutant named *epidermal cell wall1* (*ecw1*). The *ecw1* phenotype was identified in a screen of an ethyl methanesulfonate (EMS) mutagenized population for mutants that affected the changes in cell wall biochemistry which distinguish juvenile and adult leaf epidermal cell identity, as visualized by differential staining reaction with TBO (Fig. 2). The first 5–6 juvenile leaves of wild-type plants stain purple and possess a weakly- invaginated epidermal lobes. In contrast, subsequent adult leaves stain aqua with TBO and exhibit extensive lobing that interlocks the adjacent intercostal cells. The juvenile leaves of the *ecw1* mutant stain purple and are weakly-invaginated as in wild-type; however, these features also continue to persist in all adult leaves of the *ecw1* mutant.

**Figure 3:**
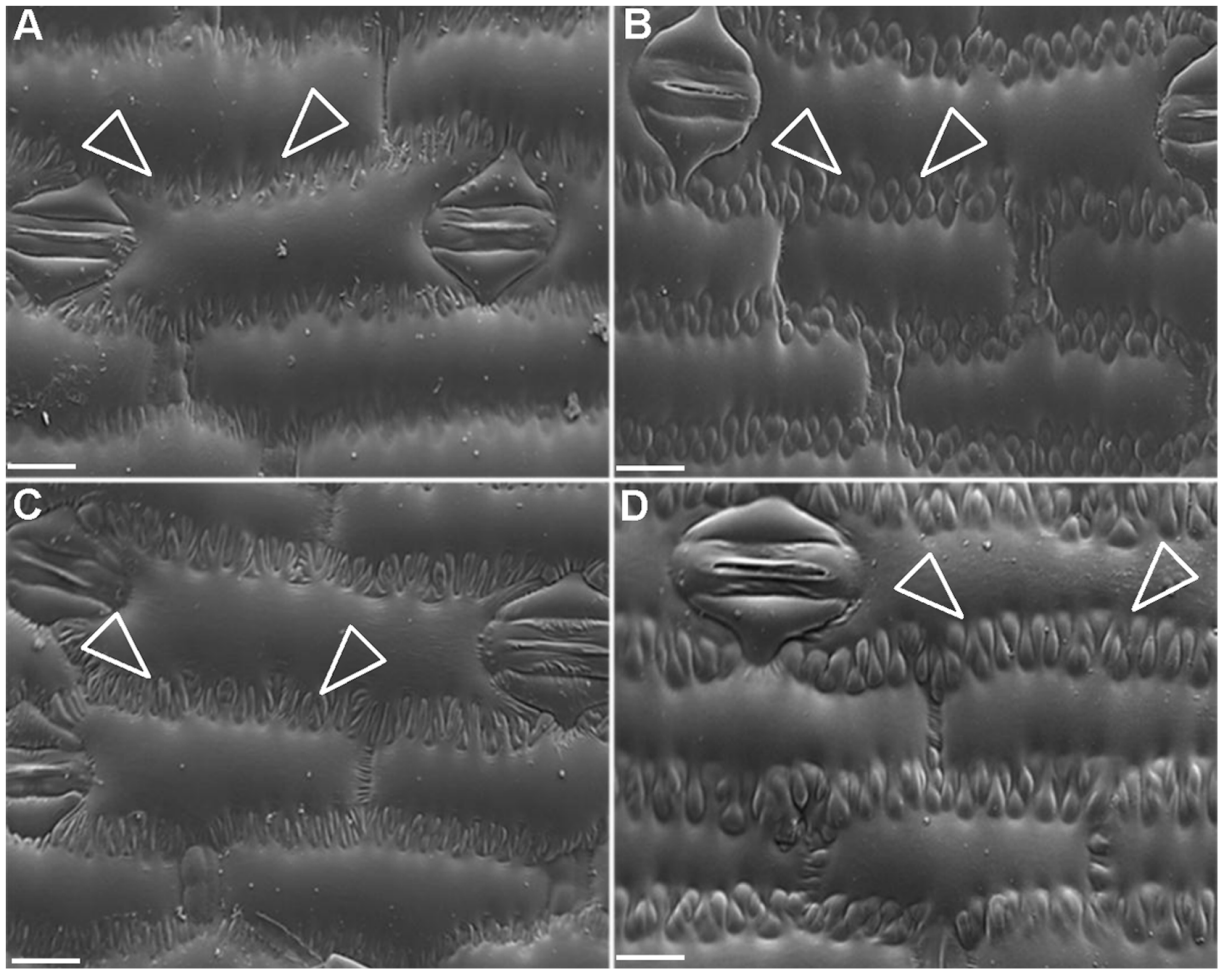
Epidermal lobing patterns in *crw1* and wild-type leaves. Representative cryoscanning electron micrographs comparing epidermal lobing pattern (indicated by arrowheads) in *crw1* leaf 7 (**A**), wild-type leaf 7 (**B**), *crw1* leaf 13 (**C**) and wild-type leaf 13 (**D**). Bar = 40 µm.

Considering the fact that maize mutants exceptionally susceptible to the WCR beetles were never reported before, we considered it likely that *ecw1* may be another mutant allele of *crw1*. To test this hypothesis, we checked the TBO staining and epidermal lobe pattern of *crw1*. We found that the TBO staining and epidermal lobing pattern of *crw1* was identical to *ecw1* (Fig. 2 and 3). We then crossed *crw1* and *ecw1* (introgressed into the B73 inbred background) to test for genetic complementation. Although the resulting F_1_ appeared as robust as the normal B73/W22 hybrid, its adult leaves stained purple with TBO as observed for *ecw1* and *crw1*. In addition, it was highly susceptible to the WCR beetles, clearly demonstrating that *crw1* and *ecw1* are allelic and result from mutations in the same gene. As a result, *ecw1* is now designated as *crw1-4*.

**Figure 4:**
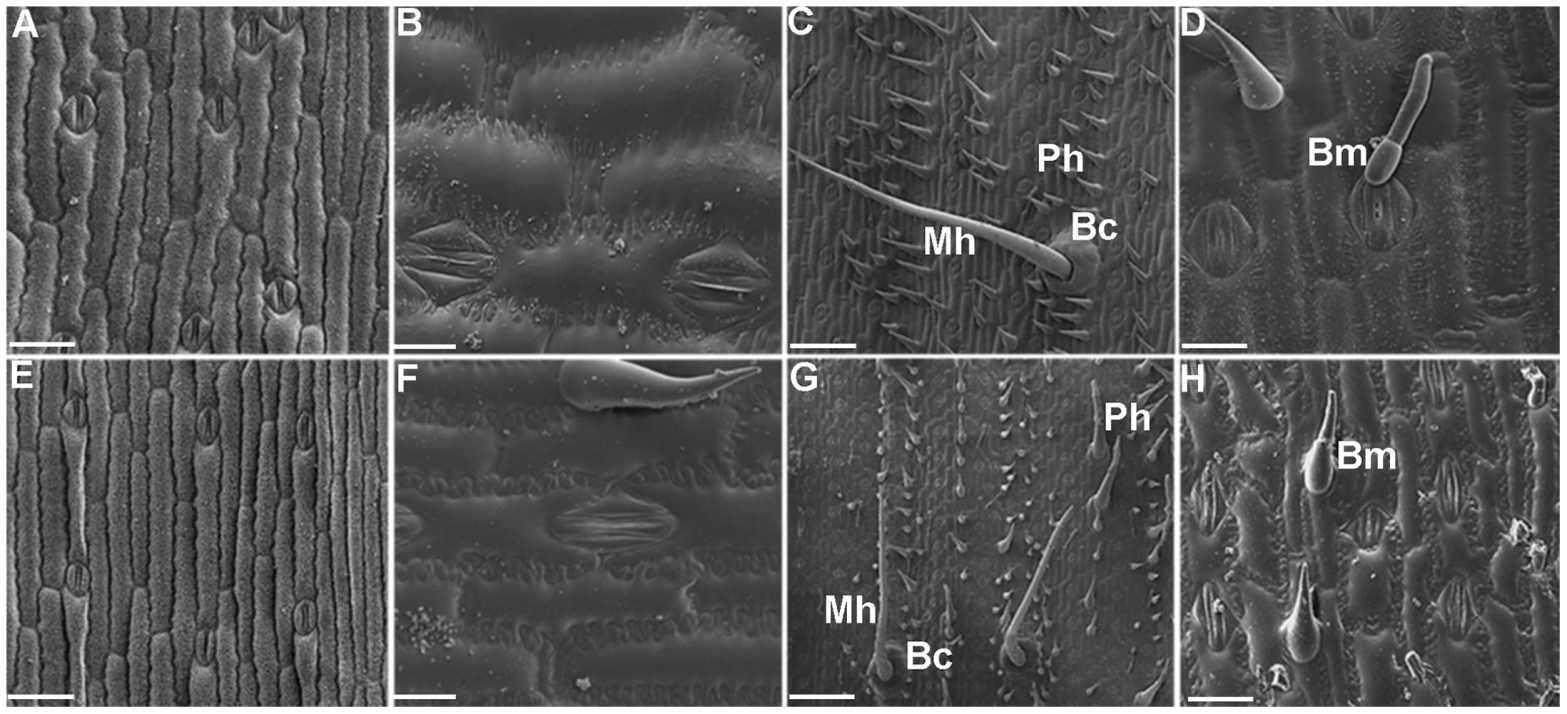
Representative cryoscanning electron micrographs comparing leaf epidermis in *crw1* and wild-type leaves. Adaxial surface of leaf 3 of *crw1* (**A**) and wild-type (**E**), covered with crystalline epicuticular waxes. Adaxial surface of leaf number 7 of *crw1* (**B**) and wild-type (**F**) showing amorphous epicuticular waxes. All four different cell types of trichomes-prickle hair (Ph), macrohair (Mh), bicellular microhair (Bm) and bulliform cells (Bc) are present both in *crw1* (**C** and **D**) and wild-type (**G** and **H**) leaf 7. Bars A and E = 100 µm, B and F = 40 µm, C, D, G and H = 300 µm.

Importantly, the *crw1* mutants were not impaired in the replacement of juvenile waxes or production of macrohairs (Fig. 4), two other leaf identity traits associated with vegetative phase change [26]. To further assess its relative position within the vegetative phase change pathway, the *crw1-4* mutation was crossed to the *glossy15* (*gl15*) mutation that coordinately regulates the entire suite of juvenile versus adult leaf epidermal traits [26]. The F_1_ plants produced fully normal juvenile and adult leaves as in wild-type plants, indicating *crw1* was not allelic to *gl15*. Self-pollination of the F_1_ plants produced an F_2_ population of 182 plants with four phenotypic classes in proportions consistent with two independently segregating recessive alleles (9∶3∶3∶1, X^2^, *p*>0.05, 3 d.f.): 112 fully wild type, 30 typical *glossy15* phenotype where all adult epidermal cell traits appear earlier at leaf 3, 32 *crw1* type with prolonged purple TBO staining but otherwise normal vegetative phase change, and 8 *crw1*; *gl15* double mutants that exhibited accelerated onset of glossy leaf wax phenotype and macrohairs at leaf 3, but purple TBO staining and weakly-invaginated walls in all leaves. These observations suggested that *crw1* is required for the biochemical features that define adult leaf epidermal cells, and indicated that the cell shape differences between juvenile and adult leaf epidermal cells are linked to these same biochemical changes.

**Figure 5:**
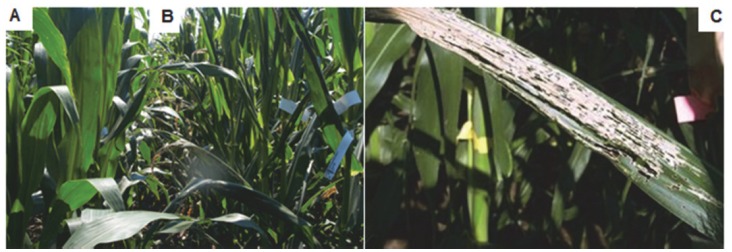
Phenotypic impacts of the *crw1-4* mutation in the B73 × Mo17 hybrid. Photograph of adjacent field plots planted with the B73×Mo17 hybrid (**A**) and the *crw1-4* near isogenic mutant hybrid (**B**) displaying extensive WCR beetle feeding and less rigid leaf architecture. **C**, Close up view of severe damage from foliar feeding by WCR beetles in *crw1-4*.

Near-isogenic lines were created for the *crw1-4* by backcrossing to both the B73 and Mo17 inbreds. The susceptibility to foliar feeding and TBO staining phenotypes were very similar in both genetic backgrounds. Because maize is grown commercially as hybrids and tolerance to root damage by WCR larvae differs between inbreds and hybrids [33], we produced the B73×Mo17 hybrids from the near-isogenic lines for *crw1-4* and evaluated them for foliar feeding, root damage ratings, and grain yield (Fig. 5, Table 1). A near-isogenic hybrid for the *gl15* mutation was also included for comparison. The leaves of the *crw1-4* hybrid showed much greater damage from foliar WCR beetle feeding compared to either the normal or *gl15* mutant hybrids (Fig. 5). There were no significant differences in root damage rating among the three hybrids and the *crw1-4* hybrid also produced approximately 30% less grain (Table 1) compared to the normal or *gl15* mutant hybrids. These observations suggest that *crw1* primarily affects foliar feeding by WCR adult beetles, and its pleiotropic effects on leaf development can reduce grain yield under moderate levels of WCR infestation.

**Table 1:** Damage by WCR larvae and adult beetles on maize hybrids.

Genotype	Foliar feeding rating^a^	Root damage score^b^	Grain yield (g/plant)
B73×Mo17	2.18+/−0.04	0.88+/−0.25	109.9+/−17.1
B73×Mo17: *gl15*	2.38+/−0.06	1.06+/−0.28	117.5+/−15.5
B73×Mo17: *crw1-4*	8.15+/−0.07	0.95+/−0.27	76.1+/−12.2

a Visual rating for proportion of tip 25 cm from leaf subtending ear, on a scale of 1 = no feeding damage to 10 = complete defoliation.

b Visual rating of number of nodes showing pruning from corn rootworm beetle feeding, on a scale of 0 to 3 full nodes pruned.

### Reduced Levels of Hydroxycinnamic Acids and Lignin in *crw1* Leaf Walls

**Figure 6:**
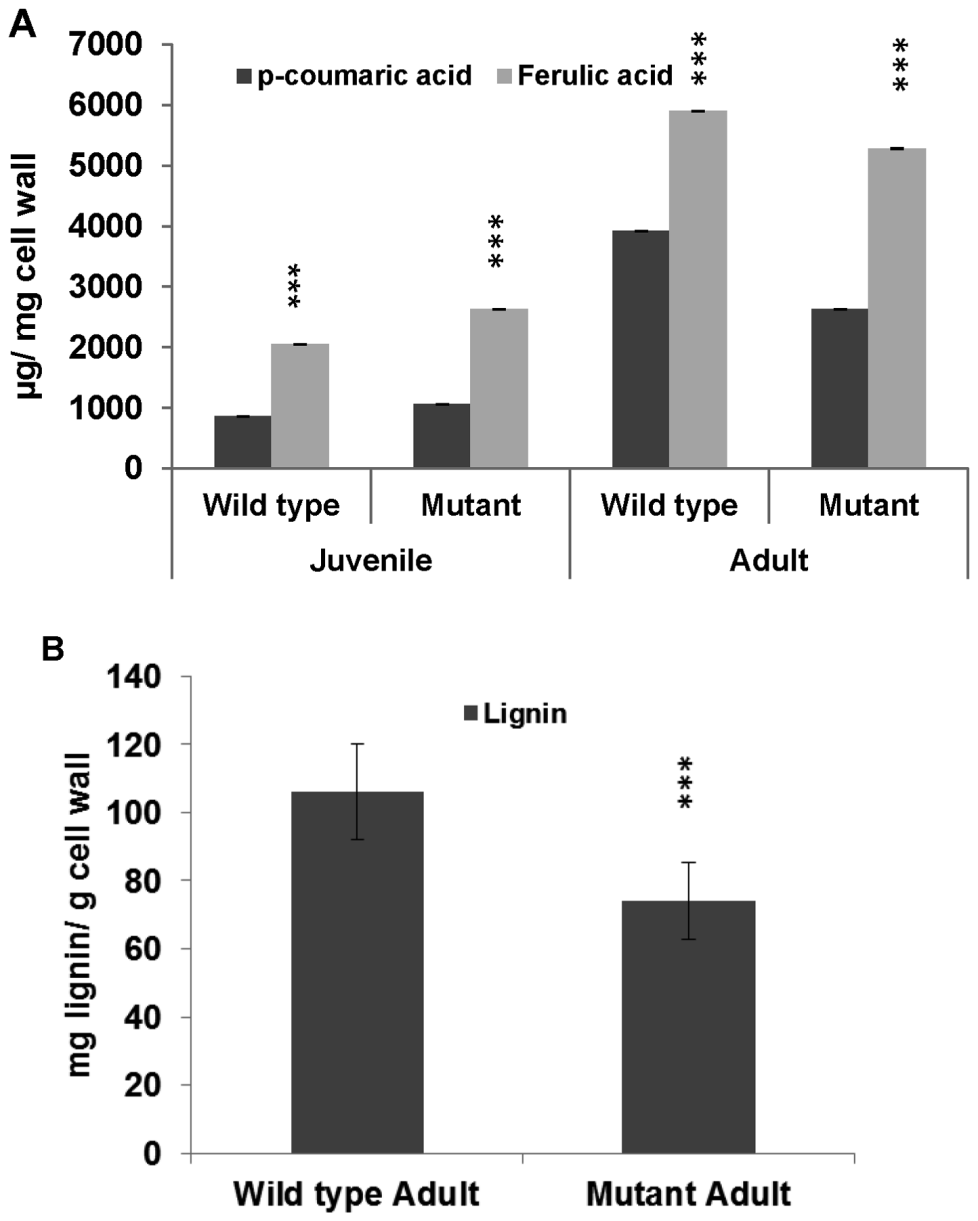
Comparison of cell wall bound hydroxycinnamic acids and lignin content isolated from Wild type and *crw1* (Mutant) foliage. **A**, differences in foliar p-coumaric and ferulic acid levels from juvenile and adult stages of Wild type and Mutant. **B**, differences in lignin content of adult leaves of Wild type and Mutant. Each data point represents the mean ± standard deviation of 3 and 9 biological samples for hydroxycinnamic acids and lignin, respectively. Asterisks denote significant differences between Wild type and Mutant (unpaired *t* test: P<0.001).

What could be the reason for the adult leaves of *crw1* to show a staining reaction with TBO like those of juvenile leaves? The polychromatic staining of TBO depends on the density of charged functional groups in cell wall components, with purple staining indicative of a higher density of reactive groups [34]. A likely source of differential staining reactions with TBO in the maize leaf epidermis is the altered accumulation of cell wall bound phenolic compounds [35], which are key determinants of TBO staining in other species [34], [36], and which are important constituents of adult grass cell walls. Two key phenolics in this regard are *p*-coumaric acid (pCA) and ferulic acid (FA), which serve to cross-link cell wall polysaccharides and lignins [37]. It had been found previously that leaves with adult identity, including the precociously adult leaves of the *glossy15* mutation, exhibit higher concentrations of pCA and FA [35]. To address if the levels of pCA and FA were compromised in *crw1*, we isolated these hydroxycinnmates from the juvenile (V3 stage) and adult (V8) walls of both *crw1* and wild type leaves by saponification of the isolated cell walls and analyzed them by HPLC. Not too surprisingly, minor variation could be detected in the total contents of pCA and FA in the juvenile leaves of *crw1* and wild type leaves. However, substantial reductions in the amounts of these cell wall bound hydroxycinnmates especially, pCA was found in the adult leaves of *crw1* (33% reduction at p<0.001; unpaired *t* test) compared to wild -type plants (Fig. 6A).

Given the intimate association between the accumulation of pCA and lignification in grasses [37]; we tested whether there were differences in the levels of lignin in the adult walls of *crw1* leaves compared to their wild-type counterparts. To address this likelihood, lignin was extracted from isolated cell walls as acetyl bromide soluble (ABS) fraction and analyzed by UV spectroscopy. Consistent with the reduction in the levels of pCA in *crw1* adult leaf walls, the levels of ABS lignin are lower (p<0.001; unpaired *t* test) in the *crw1* leaves at maturity compared to wild-type leaves (Fig. 6B). The *brown midrib* (*bm*) mutations of maize also exhibit reductions in lignin and changes in pCA, FA and their esterification [38], yet do not suffer from enhanced susceptibility to foliar feeding by WCR beetles or exhibit altered TBO staining. Similarly, although the *crw1* mutant shows reductions in lignin, they do not show the brown midrib phenotype, indicating *crw1* affects cell wall phenolics via a distinct genetic pathway. This hypothesis was confirmed by the double mutant analysis of *crw1-4* in combination with each of the *bm1*, *bm2*, *bm3* and *bm4* mutations. Within each population of F_2_ progeny produced from the cross of *crw1* with a *brown midrib* mutation, the segregation ratios for single and double mutant phenotypes were consistent with two independently-acting recessive mutations. Approximately 1/16 of the individuals produced completely normal juvenile leaves and adult leaves that possessed both reddish-brown midribs and that stained uniformly purple with TBO.

### Reduced Tensile Strength of *crw1* Leaves

**Figure 7:**
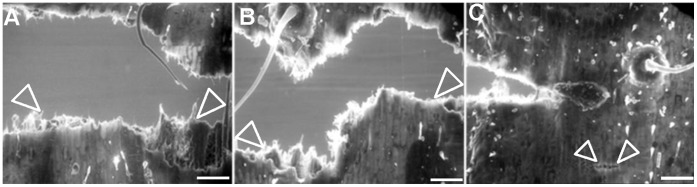
Representative uni-axial tensile stage scanning electron micrographs comparing fracture dynamics of wild-type and *crw1* adult leaves. The incipient crack propagation profile and breakage dynamics at the point of final failure of wild-type (**A**, area between arrowheads) and *crw1* (**B**, area between arrowheads). **C**, Additional fracture points along the axis of incipient crack propagation in *crw1* (area between arrowheads). Bar = 200 µm.

The adult *crw1* leaves were found to be compromised in three strength-imparting components of cell walls, namely the epidermal cell invaginations, cellulose tethering hydroxycinnamates, and lignification. Accordingly, *crw1* leaves appear to be less rigid than wild-type. We compared tensile strength of *crw1* and wild-type leaves with a tensile stage SEM. The results obtained indicated that there are significant differences in both crack propagation and fracture dynamics between *crw1* and wild type leaves. While adult leaves of wild type plants from any position along the leaf lamina form even fracture surfaces, equivalent *crw1* leaves form uneven fractures when pulled to the point of final failure (Fig. 7A and B). Moreover, *crw1* leaves tend to form additional fractures along the axis of the incipient crack propagation (Fig. 7C). Because the formation of uneven and multiple fracture lines are often associated with low structural integrity in plant tissues [39], these results indicate that *crw1* leaves have a lower tensile strength compared to wild-type leaves.

### *Crw1* Maps to Chromosome 6

A population of 441 plants segregating for the *crw1-4* allele in the B73 inbred background were scored for the *crw1* mutant phenotype and used to obtain a genetic map position for the *crw1* locus. As expected for a single recessive locus, approximately 25% (118) of these individuals exhibited the *crw1* mutant phenotype. Pools of wild type and *crw1-4* mutant plants were used in a bulk segregant analysis approach and genotyped with 384 SSR markers selected to uniformly span the maize genetic map. Five markers (*bnlg161*, *gpc2*, *umc1753*, *bnlg1867*, *umc1229*) located to chromosome bin 6.00 on the IBM Neighbors2 map were found to exhibit differential amplification among the wild-type and *crw1-4* mutant pools, indicative of segregation distortion and genetic linkage. Further genotyping of 112 *crw1-4* mutant individuals with these five markers refined the genetic map in this region and located *Crw1* in a 17 centiMorgan interval between *bnlg161b* (8 recombinants) and *gpc2* (12 recombinants). *In silico* mapping of these flanking markers on the B73 reference genome sequence (www.maizesequence.org) indicated this chromosomal region spanned nearly 6 Mbp.

## Discussion

We describe here the discovery, mapping and preliminary characterization of *crw1,* a corn mutant that was identified on the basis of its exceptional foliar susceptibility to WCR beetles. Its recessive loss-of-function nature suggests that a key characteristic which is usually required for deterring WCR beetles is lacking in this mutant. It is also possible that this mutation leads to perturbations in biochemical pathways resulting in the accumulation of pathway intermediates that might enhance the attraction of corn foliage to WCR beetles. One curiosity is that this mutant was never reported before, perhaps because the most dramatic visual aspect of the *crw1* phenotype is conditional upon sufficient WCR beetle feeding pressure.

Although the adult hosts of WCR beetles are varied [19]–[21], extensive feeding (to the point of total defoliation) upon corn foliage is very unusual. The WCR beetles primarily feed on corn pollen and silks but target the relatively nutrient-deficient leaves only as a last resort [19]. WCR beetles are known to survive on pollen of alternate hosts (mostly weeds) during first few weeks of their adult life, until corn pollen and silks becomes available [40]. They show very low preference to late vegetative or reproductive stage corn foliage [22]–[23]. The continued feeding upon *crw1* foliage by WCR beetles starting from early adult vegetative stage (V5–V6 stage) through reproductive maturity (Fig. 1A and B, left panel) is a stark deviation from the norm. This unusual feeding choice demonstrates that an active deterrence mechanism might be present in corn foliage which restricts the feeding preference of WCR beetles to silks and pollen. We hypothesize that the absence of this active deterrence mechanism in *crw1* leads to its exceptional foliar susceptibility.

The onset of foliar susceptibility in *crw1* coincided with vegetative phase change, and the purple TBO staining and moderate lobing of *crw1* leaf epidermal cells (Fig. 2 and 3) suggested that *crw1* may function in the transition from juvenile to adult leaf identity. Interestingly, there have been previous reports of enhanced susceptibility to insect herbivory when the juvenile phase and purple TBO staining of leaf epidermal cells is prolonged by the maize *Corngrass1* mutation [41]–[44] that overproduces *microRNA156* (45). However, except perhaps for *Corngrass1* (*Cg1*), none of the other known maize mutants that alter vegetative phase change (*Teopod1* (*Tp1*), *Teopod2* (*Tp2*), GA-deficient or insensitive loci, *early phase change1* (*epc1*), or *glossy15 (gl15)*, exhibit enhanced susceptibility to foliar feeding by WCR (Table 1, S. Moose, Unpub.). Conversely, *crw1* only affects TBO staining and cell shape but not other traits associated with vegetative phase change (Fig. 4). Double mutant analysis indicated that *Crw1* function is required for the accelerated onset of aqua TBO staining in leaves 3–7 of *glossy15* mutants. These observations indicate that *Crw1* promotes the biochemical and morphological features of intercostal cells within the epidermis of normal adult leaves, and may be repressed by *glossy15* in normal juvenile leaves. Another possibility for the late onset of foliar susceptibility in *crw1* might be the DIMBOA pathway. The DIMBOA pathway confers early stage resistance to insects in corn however, the potency of this resistance declines with age [45]. However, double mutant analysis of *crw1* with *bx1,* a mutant impaired in DIMBOA pathway, indicated no link between DIMBOA with the foliar susceptibility conditioned by *crw1* (BP Venkata, Unpub.). These observations are consistent with the recent findings that WCR larvae are completely immune to the products of the DIMBOA pathway [46].

The altered TBO staining pattern of the adult *crw1* intercostal cells might be due to reduced levels of cell wall bound phenolic acid monomers (pCA and FA), which appear to be critical determinants of the staining pattern [34]–[36]. Consistent with this hypothesis, the levels of pCA are substantially lower in adult *crw1* leaves compared to the wild type (Fig. 6A). Further, since pCA is esterified to the γ position of S lignin subunit in grasses, its accumulation is usually considered to be a marker for lignin deposition [37], [47], [48]. Not surprisingly, *crw1* shows a reduction in foliar lignin content during adult stages (Fig. 6B). In general, higher levels of lignin and cross linking of cell wall components by cell wall bound phenolic compounds enhance both leaf and stalk stiffness in grasses [37]. In addition, there have been previous reports correlating increased phenolic acid and lignin levels with resistance to insect pests in corn [37], [49]. Despite this, none of the corn *bm* mutants (*bm1, bm2, bm3* and *bm4*) with altered lignin content and/composition [38] exhibit unusual foliar susceptibility to WCR beetles. Interestingly, the percentage reduction of lignin in *crw1* is comparable to the overall reduction in the lignin levels of *bm2* and *bm3* [50], [51], negating the exclusive role of reduced lignin content in its foliar susceptibility to WCR beetles. Further, none of the *bm* mutants of maize shared the altered TBO staining phenotype with *crw1*, nor do *crw1* mutations exhibit a brown-midrib phenotype. Therefore, our finding that the *crw1* mutation acts in an independent genetic pathway from the *bm* mutants indicates that the impacts of *crw1* on maize cell wall phenolics may be mediated by changes in either the degree of cross-linking among hydroxycinnamic acids or between these phenolics and the glucoronoarabinoxylans that comprise the major carbohydrate polymer in maize leaf cell walls. Consistent with the above, the 17 centiMorgan interval on chromosome 6 to which *Crw1* maps is devoid of any known genes or regulators for phenylpropanoid biosynthesis.

In grass species like corn the intercostal cell lobes function to enhance intercellular adhesion through physical interlocking [52]. The moderate epidermal lobing pattern in c*rw1* in conjunction with reduced levels of cell wall components including lignin might contribute towards its lower leaf toughness during the adult stage. The results from uniaxial tensile stage SEM support this hypothesis. The dynamics of incipient crack extension in the adult *crw1* leaves is suggestive of lower resistance around the plane of crack extension that resulted in an uneven fracture surface at the point of critical failure (Fig. 7B). This is in agreement with previous reports that associate this characteristic with lower structural integrity [39]. The development of additional fracture lines along the axis of the incipient crack propagation in the adult *crw1* leaves (Fig. 7C) further substantiates the above argument. There is a strong body of evidence that leaf mechanical properties, including strength and toughness, have a detrimental effect on insect herbivory [37], [53], [54]. The mechanical properties leading to leaf toughness might deter insect herbivory either by mechanical constraints or by dilution of the nutrients [55], [56]. Therefore, the lower structural integrity of *crw1* leaves during the adult stage might be conducive for preferential feeding by WCR beetles. However, we also note that other maize genotypes that display ultrastructural compromise, such as the *brittle stalk* (*bk2)* mutation [57] or populations with low rind penetrometer resistance do not show enhanced susceptibility to foliar damage by WCR beetles. Similarly, although the intercostal cell lobing pattern observed for *crw1* is identical to the *Extra cell layer (Xcl1)* [58] mutant, neither *Xcl1* nor the *brick1* (*brk1*) or *brick2* (*brk2*) mutants that completely lack intercostal cell lobes [59] display enhanced foliar susceptibility to WCR beetles.

### Conclusion

Taken together, the data presented here indicates that the *crw1* mutation causes pleiotropic defects on leaf epidermal cell wall biochemistry and shape, yet defines a genetic pathway that contributes to resistance to foliar feeding by the WCR beetle. It does not appear that any single perturbation of leaf epidermal cell walls is specifically important for the foliar susceptibility to WCR beetle, suggesting that this feature of the *crw1* mutant phenotype is a combinatorial effect of reduced lobes, lowered lignification and altered cell wall composition as indicated by changes in TBO staining. In such a scenario, it should be possible to reconstitute the WCR foliar susceptibility in a triple mutant of *gl15* (a phase transition mutant), *bm2* (lignification mutant) and *brk1* (a mutant devoid of epidermal cell lobes). The generation of such triple mutant is currently underway. It is also possible that other mechanisms in addition to cell wall biochemistry may contribute to the enhanced susceptibility of *crw1* leaves to WCR feeding. The discovery of *crw1* opens up a new avenue to identify native resistance to WCR that might significantly mitigate the economic scourge caused by this insect pest.
